# Supplementary material for: Mobile barrier mechanisms for Na+-coupled symport in an MFS sugar transporter
Source: eLife. 2024 Feb 21;12:RP92462. doi: 10.7554/eLife.92462 (PMC10942615; doi:10.7554/eLife.92462)
Supplement: Figure 1—source data 1. [file elife-92462-fig1-data1.pdf]

Figure 1 – Source Data 1

In vivo two-hybrid interaction assay in *E. coli* DH5α *cyaA* strain

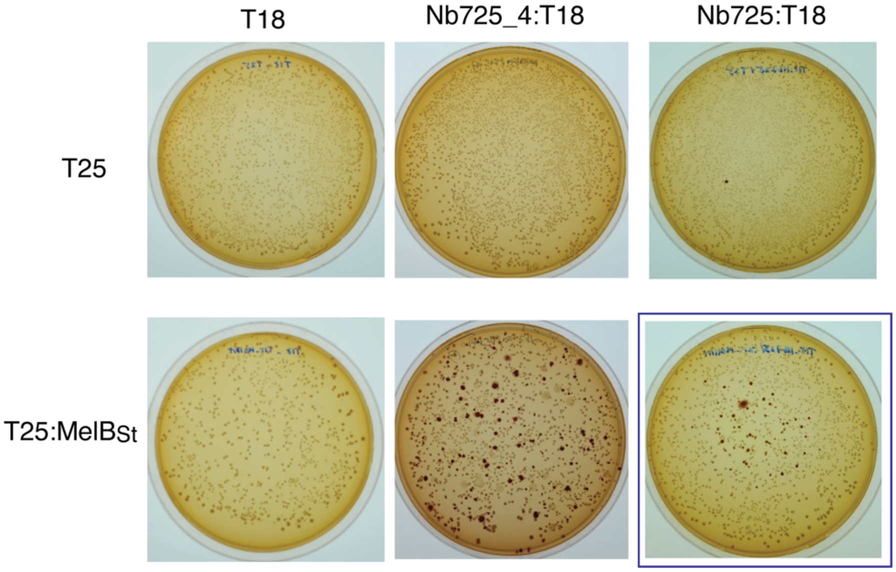

Figure 1 – source data 1. In vivo two-hybrid interaction assay. Two compatible plasmids derived from pACYC and pCS19 encoding T25:$MelB_{St}$ and Nb:T18, respectively, were transformed into E. coli DH5*α* cyaA cells and plated on the maltose-containing MacConkey agar plate as described in Methods. The irregular red colonies are typical of a positive test indicating a protein-protein interaction. The Figure 1a was cropped from above images. The image for the two hybrids T25:$MelB_{St}$ and Nb725:T18 as boxed in blue was re-used from a JBC paper figure 3a[29] (https://doi.org/10.1016/j.jbc.2023.104967). Creative Commons Attribution (CC BY 4.0) |
