# Supplementary material for: Mobile barrier mechanisms for Na+-coupled symport in an MFS sugar transporter
Source: eLife. 2024 Feb 21;12:RP92462. doi: 10.7554/eLife.92462 (PMC10942615; doi:10.7554/eLife.92462)
Supplement: Figure 1—source data 2. [file elife-92462-fig1-data2.pdf]

Figure 1 – Source Data 2

Nbs specifically inhibite melibiose fermentation in *E. coli* DW2 strain

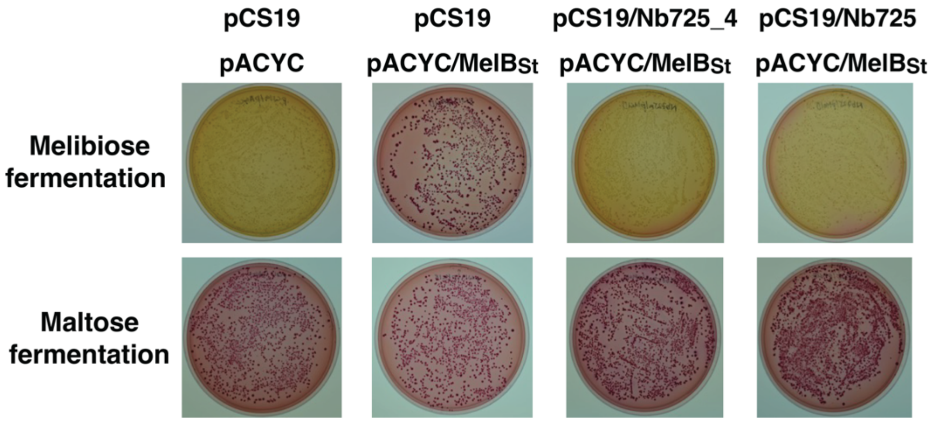

Figure 1 – source data 2. Sugar fermentation assay. Two compatible plasmids derived from pACYC and pCS19 encoding $MelB_{St}$ and Nb725 or Nb725_4, respectively, were transformed into *E. coli* DW2 cells [Δ*melB*Δ*lacYZ*] and plated on the MacConkey agar plate containing maltose (for the positive control) or melibiose (for testing the transport activity of $MelB_{St}$) as the sole carbon source. Magenta: strong sugar fermentation; yellow: no sugar fermentation.
