# Supplementary figures and images for: Mobile barrier mechanisms for Na+-coupled symport in an MFS sugar transporter
Source: eLife. 2024 Feb 21;12:RP92462. doi: 10.7554/eLife.92462 (PMC10942615; doi:10.7554/eLife.92462)

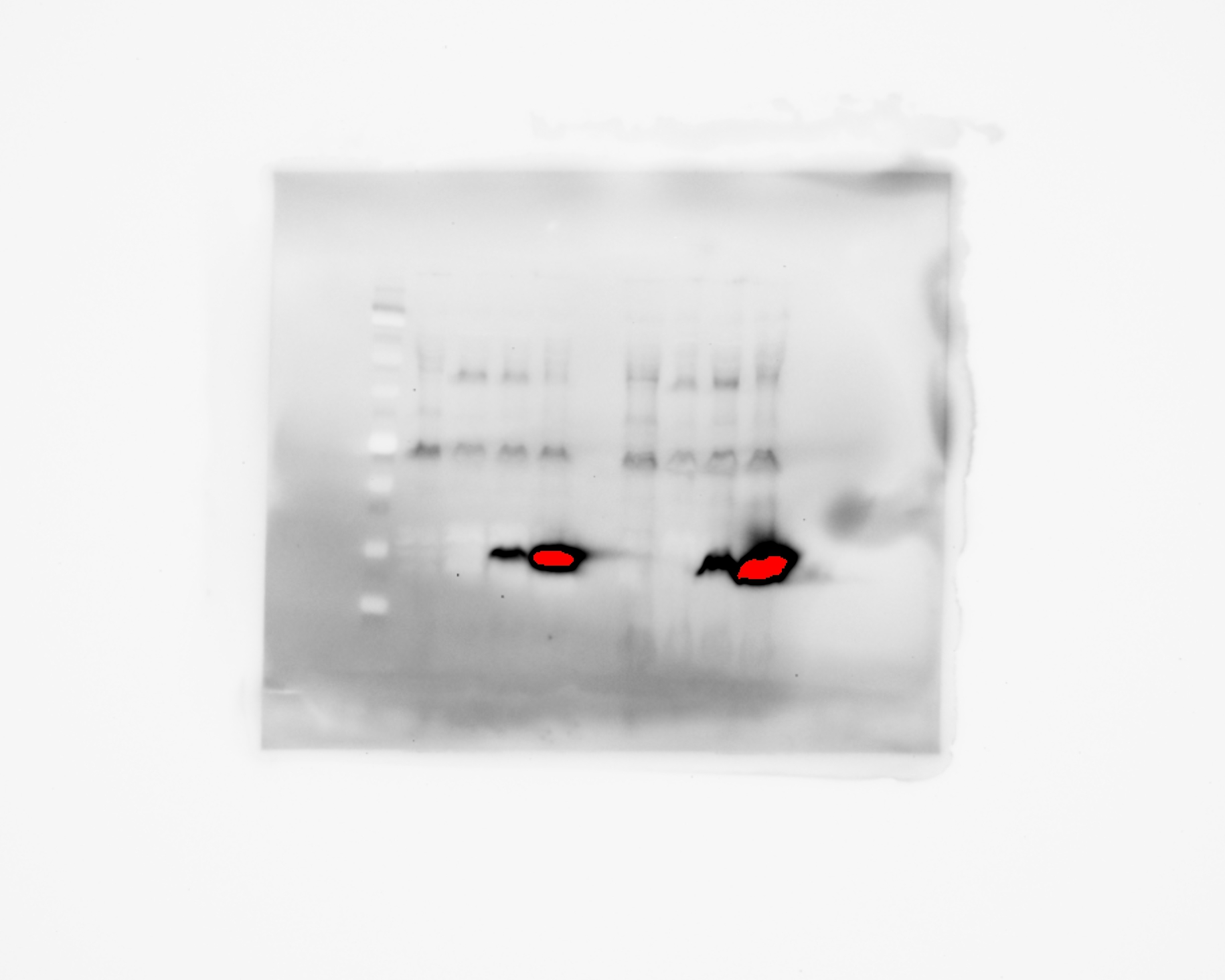

Supplement: Figure 1—source data 3. — Membranes were prepared from E. coli DW2 cells that were transformed with two compatible plasmids derived from pACYC and pCS19 encoding MelBSt and Nb725_4 or Nb725, respectively, and used for the [3H]melibiose active transport assay. After protein concentration determination, 50 μg of total membrane proteins of each sample was analyzed by SDS-15%PAGE and western blot using anti-His tag antibody (HisProbe-HRP Conjugate) as described in the Materials and methods. The western blot result was imaged by the ChemiDoc MP Imaging System (Bio-Rad). MelBSt protein expression presented as the inset in Figure 1c. The bands migrating near 25 kDa are non-specific and also presented in the membranes with no MelB nor Nb. [file elife-92462-fig1-data3.jpg]

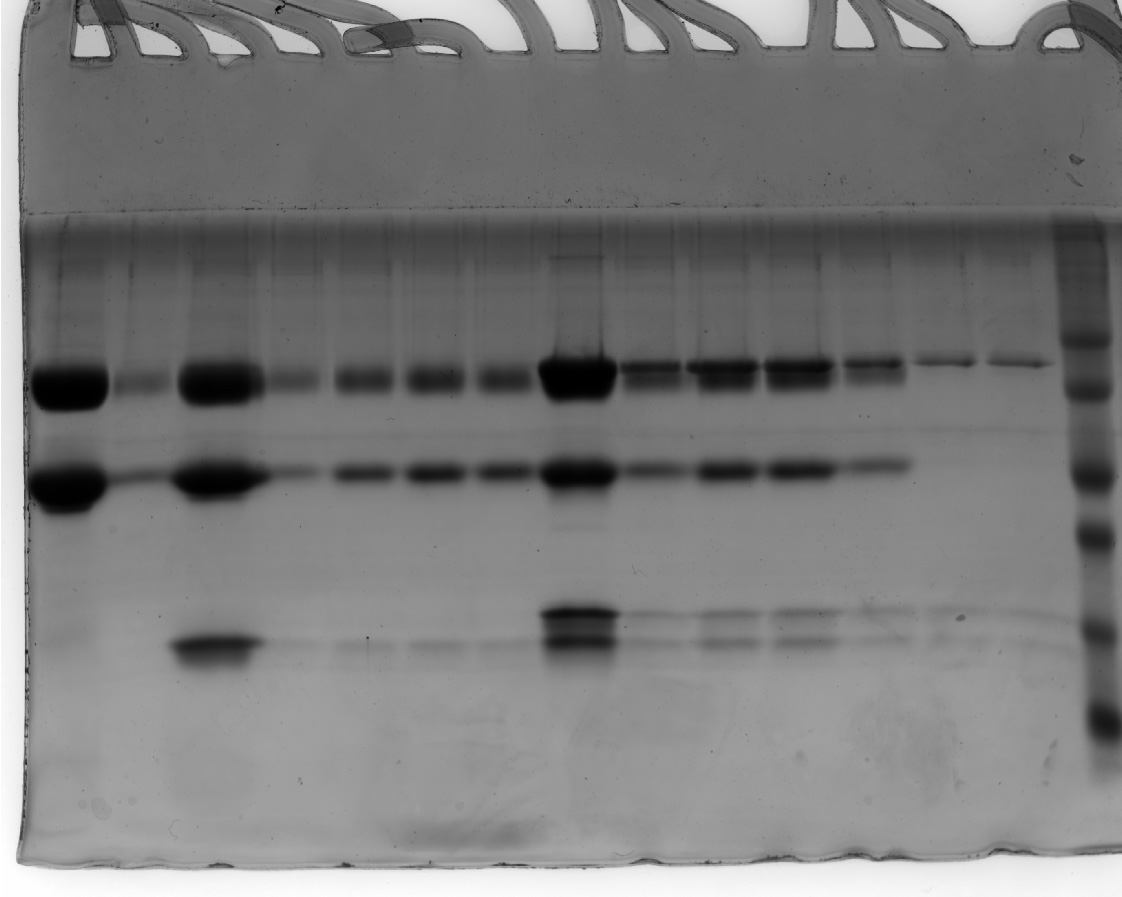

Supplement: Figure 1—figure supplement 1—source data 1. — Fractions collected from gel chromatography were analyzed by SDS-15% PAGE and stained by silver nitrate. [file elife-92462-fig1-figsupp1-data1.jpg]

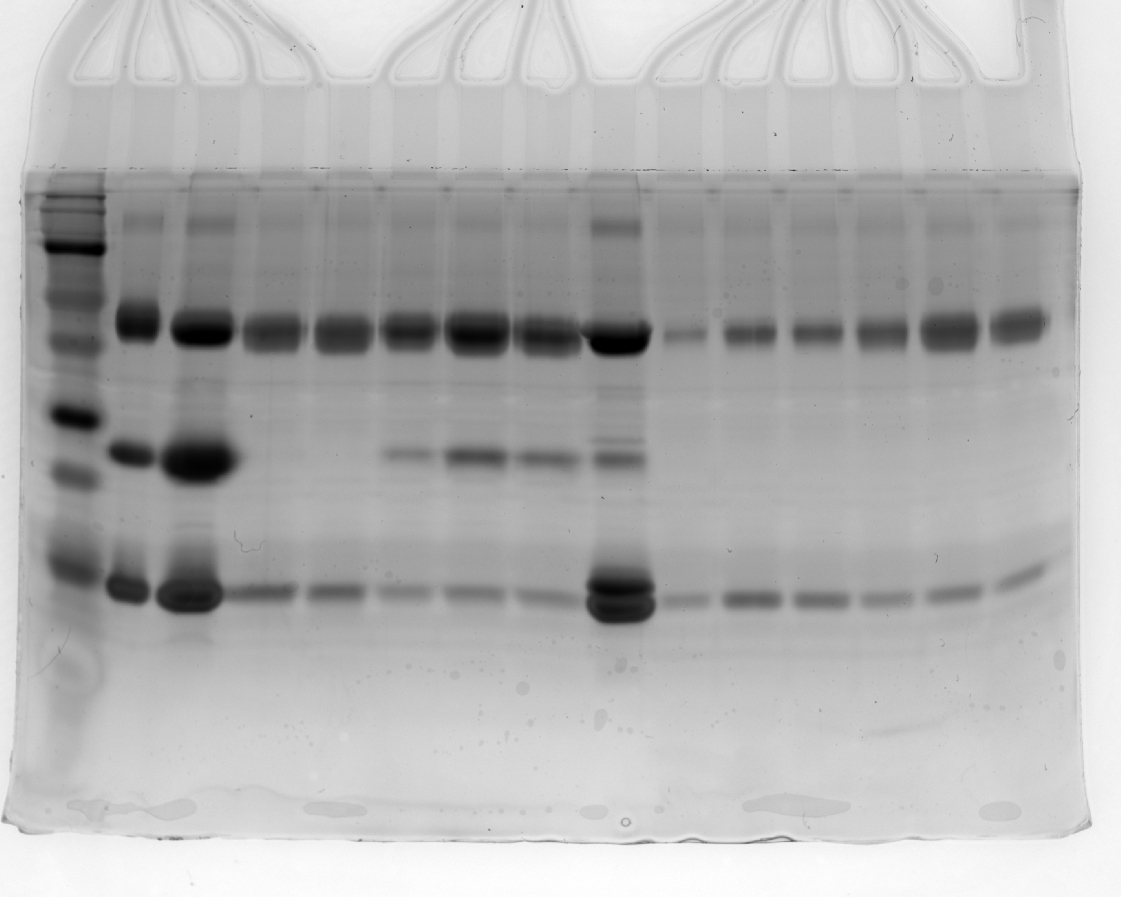

Supplement: Figure 2—figure supplement 8—source data 1. — Fractions collected from gel chromatography were analyzed by SDS-15% PAGE and stained by silver nitrate. [file elife-92462-fig2-figsupp8-data1.jpg]
