# Supplementary material for: Mobile barrier mechanisms for Na+-coupled symport in an MFS sugar transporter
Source: eLife. 2024 Feb 21;12:RP92462. doi: 10.7554/eLife.92462 (PMC10942615; doi:10.7554/eLife.92462)
Supplement: Figure 1—source data 4. — Membranes were prepared from E. coli DW2 cells that were transformed with two compatible plasmids derived from pACYC and pCS19 encoding MelBSt and Nb725_4 or Nb725, respectively, and used for the [3H]melibiose active transport assay. After protein concentration determination, 50 μg of total membrane proteins of each sample was analyzed by SDS-15%PAGE and western blot using anti-His tag antibody (HisProbe-HRP Conjugate) as described in the Materials and methods. The western blot result was imaged by the ChemiDoc MP Imaging System (Bio-Rad). MelBSt protein expression presented as the inset in Figure 1c is highlighted by the box. The bands migrating near 25 kDa are non-specific and also presented in the membranes with no MelB nor Nb. [file elife-92462-fig1-data4.pdf]

## Figure 1 - source data 4

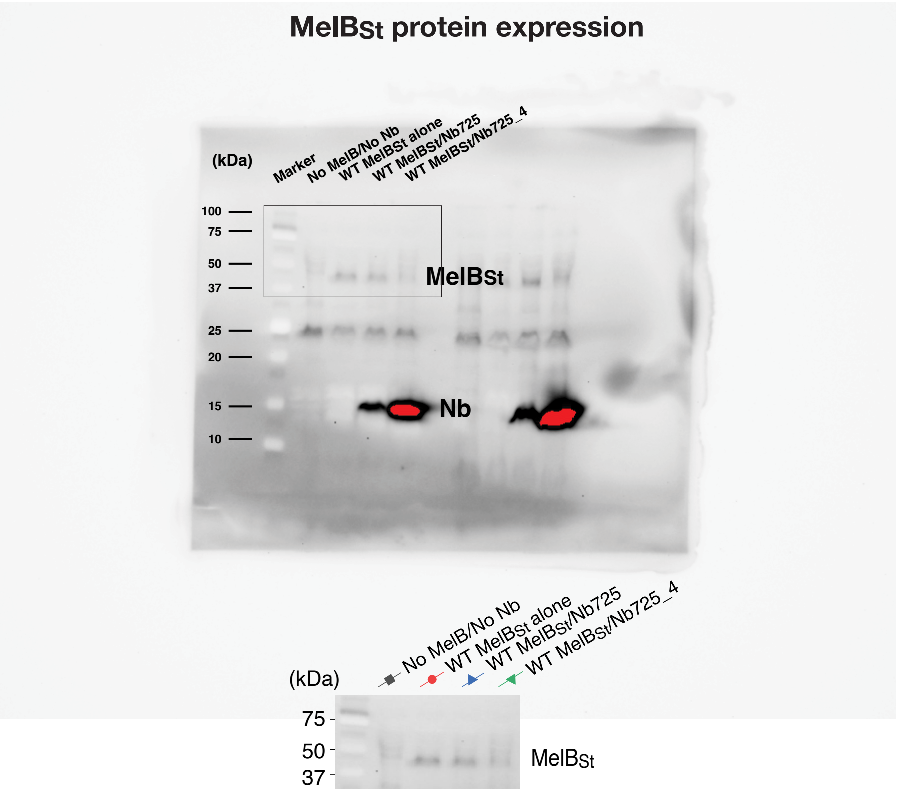


**Figure 1 – source data 4.** Western blot to detect $MelB_{St}$ expression when co-expressing with Nb725 or Nb725_4. Membranes were prepared from *E. coli* DW2 cells that were transformed with two compatible plasmids derived from pACYC and pCS19 encoding $MelB_{St}$ and Nb725_4 or Nb725, respectively, and used for the [$^3$H]melibiose active transport assay. After protein concentration determination, 50 µg of total membrane proteins of each sample was analyzed by SDS-15%PAGE and western blot using anti-His tag antibody (HisProbe™-HRP Conjugate) as described in Methods. The western blot result was imaged by the ChemiDoc MP Imaging System (Bio-Rad). $MelB_{St}$ protein expression presented as the inset in figure 1c was highlighted by the box. The bands migrating near 25 kDa are non-specific and also presented in the membranes with no MelB nor Nb.
