# Supplementary material for: Mobile barrier mechanisms for Na+-coupled symport in an MFS sugar transporter
Source: eLife. 2024 Feb 21;12:RP92462. doi: 10.7554/eLife.92462 (PMC10942615; doi:10.7554/eLife.92462)
Supplement: Figure 1—figure supplement 1—source data 2. — Fractions collected from gel chromatography were analyzed by SDS-15% PAGE and stained by silver nitrate. [file elife-92462-fig1-figsupp1-data2.pdf]

Figure 1—figure supplement 1-source data 2

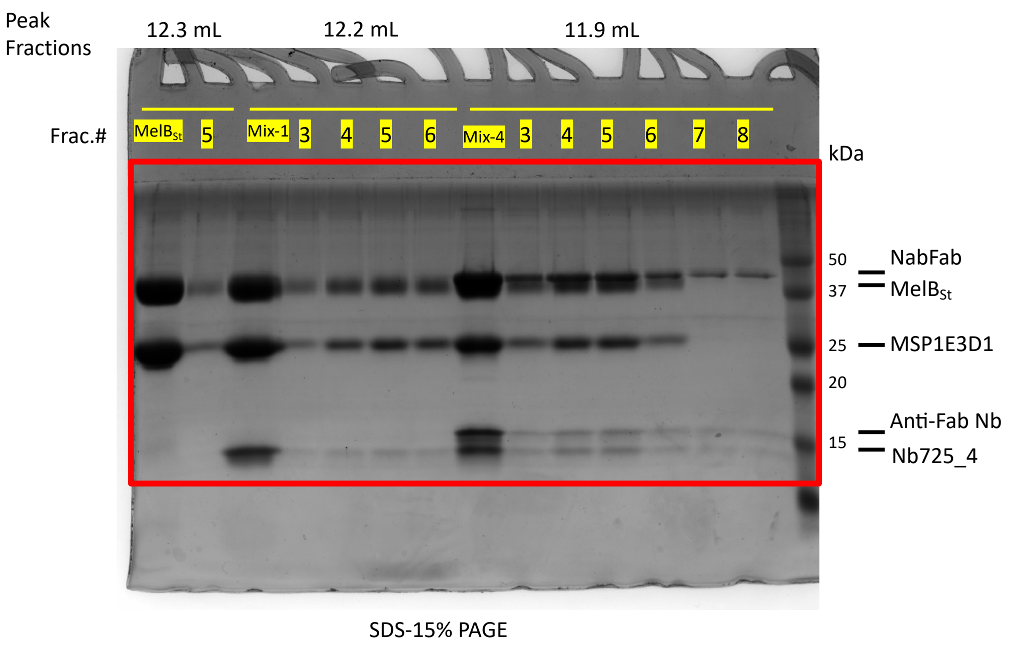

Figure 1—figure supplement 1-source data 2. Fractions collected from gel chromatography were analyzed by SDS-15% PAGE and stained by silver nitrate.
